# Indents

**Published:** 1902-03-15

**Authors:** 


### INDENTS.

**☞ Brief Articles of Technical or Scientific Value will receive notice and comment. Contributions invited.**

To Keep Hydrogen Peroxide.—Tightly cork the bottle and keep it standing inverted in a vessel of water. Small quantities may be kept out for immediate uses.—*Dominion Journal.*

Platinum Solder. — Add from two to six grains of platinum to one pennyweight of pure gold. Thoroughly fuse and roll thin. Repeat the operation of melting and rolling several times to thoroughly mix the metals.—Charles Bonsal, in *The Brief.*

Castor Oil for Neuralgia.—Dr. Harlan recommends giving one ounce of castor oil each day for three weeks as a cure for neuralgia. It is given in capsules of one dram each, taking four before breakfast and four more at intervals during the day. —*Review.*

Baby Ribbon for Contouring Amalgam Fillings. —Have on hand three or four widths of baby ribbon with smooth edges. Cut off strips of suitable length and pass between the teeth and use to contour the amalgam before it sets. It is strong and thin and will readily adapt itself to the contour of the tooth.—S. J. Spence, in *The Brief.*

Staining Artificial Teeth.—Very satisfactory artistic results in artificial dentures may be obtained by judicious grinding or re-shaping the individual teeth to imitate abrasions or natural wear; and also in staining the teeth with the paints furnished by Ash & Sons and burning them in the electric oven. The fine lines of enamel checks, tobacco stains, etc., can be very perfectly brought out with slight difficulty.—*Wm. Mitchell.*

FIRE CRACKING OF PORCELAIN CROWNS.—Dr. Gilford suggests that this is very often occasioned by the too liberal use of borax, which works its way in behind the backing. He suggests that this may be overcome by placing a piece of No. 30 gold foil between the metal backing and the porcelain tooth. It can be so closely adapted by means of a rubber cork as to preclude the possibility of any borax getting at the tooth.—*International Journal.*

TO HARDEN AND PROTECT PLASTER CASTS.—Take an ordinary cabinet-maker's glue pot and melt in the inner pot with boiling water, *stearin*. Have the plaster model perfectly dry and warm, and then place it for from five to ten minutes in the melted stearin. Remove and allow to stand until cool. It will be found that it has a marbleized appearance from the stearin, which has penetrated fully an eighth of an inch. Models treated in this way can be used for fitting clasps or bands without injury to the plaster teeth. It also keeps the models from becoming soiled.—*Wm. Mitchell*, London, Eng.

PRESERVATION OF THIRD MOLARS.—Dr. Stewart J. Spence, of Harriman, Tenn., writing on the subject of the preservation of third molars, after commenting on an editorial on the same subject, printed by us last year, says: "I write to suggest another reason for preserving the *dens sapientæ*. With the third molar gone, the tongue strikes against the linguo-distal corner of the second molar in its forward movements, and is thereby constantly irritated. The irritation is often not enough to attract immediate attention, and therein lies the danger. I had a patient who died of cancer of the tongue, brought on, I believe, by this cause. I rounded the angle of the tooth and advised her to consult a physician about that sore spot on the side of her tongue; but she believed she ought to trust the Lord to heal her of all her diseases, and fell a victim to this faith.—*Items of Interest*.

NARGOL IN EMPYEMIA OF THE ANTRUM.—There are objections to the nitrate of silver. It coagulates the albumins of the tissues forming a protective covering for underlying tissues; its action is therefore superficial in the treatment of purulent inflammations of mucous membranes. It also causes indelible stains that may be a source of annoyance to patient and physician. A silver salt that does not coagulate the albumins nor stain the tissues or clothing is an improvement. Nargol, a compound of silver and nucleinic acid is such a substance. I have found it a very efficient germicide and alterative in the treatment of three obstinate cases of empyema of Highmore's antrum. After the persistent though futile use of ordinary methods of treatment, I was able to effect cures in these cases by means of injections of solutions of Nargol having a strength of one-fourth of one percent. (0.25%).—Dr. A. C. Wippern, in *Med. News*.

